# Dietary Heterocyclic Amine Intake and Risk of Esophageal Squamous Cell Carcinoma in Rural Uganda

**DOI:** 10.23937/2378-3419/1410152

**Published:** 2021-06-30

**Authors:** Samson OKELLO, Emmanuel BYARUHANGA, Suzan Joan AKELLO, Emmanuel DWOMOH, Christopher Kenneth OPIO, Kathleen E. COREY, Ponsiano OCAMA, Jingshu GUO, Winnie R. MUYINDIKE, Robert J. TURESKY, David C. CHRISTIANI

**Affiliations:** 1 Department of Internal Medicine, Mbarara University of Science and Technology, Uganda; 2 Department of Medicine, College of Health Sciences, Makerere University, Uganda; 3 Harvard Medical School, USA; 4 Department of Medicine, Massachusetts General Hospital, USA; 5 Department of Medicinal Chemistry, University of Minnesota, USA; 6 Masonic Cancer Center, University of Minnesota, USA; 7 Departments of Environmental Health and Epidemiology, Harvard T.H. Chan School of Public Health, USA

## Abstract

Dietary exposure to 2-amino-1-methyl-6-phenylimidazo [4,5-b] pyridine (PhIP) in cooked meats maybe responsible for the high burden of Esophageal squamous cell carcinoma (ESCC) in southwestern Uganda.

We conducted a pilot case-control study among 31 histologically confirmed ESCC cases and 54 age, gender, and residence matched healthy community controls sampled from the general population at the time of accrual of each case in southwestern Uganda. We collected data including smoking, alcohol consumption, diet, and scalp hair samples analyzed for normalized PhlP (adjusted per gram of melanin). We used logistic regression to determine the association of PhlP and ESCC.

Overall, the mean normalized PhIP (ng/g melanin) was 44.79 (SD 148.08), higher among women compared to men (130.68 vs. 9.00, p = 0.03), lowest among healthy men [8.31 (SD 8.52) ng/g melanin] and highest among healthy women 158.39 (SD 288.75) ng/g melanin.

In fully adjusted models, covariates associated with greater odds of ESCC included ever smoking 2 to 3 pack years of cigarettes (aOR 7.75 (95% CI 1.90, 31.50) and those 3 or more pack years (aOR5.82, 95%CI 1.25, 27.11), drinking 3 to 4 alcoholic drinks daily (aOR8.00, 95%CI 2.31, 27.74), and normalized PhIP above 75th percentile (8.65 ng/g of melanin) (aOR4.27, 95%CI 1.12, 16.24).

In conclusion, high PhIP levels maybe associated with ESCC in a rural Uganda, a high ESCC burden setting. Further study with larger sample with a wider geographical representation is needed to validate scalp hair PhIP for assessment of ESCC risk.

## Background

In Asia and East Africa where Esophageal Squamous Cell Carcinoma (ESCC) - a subtype of esophageal cancer that accounts for at least 80 % of all global esophageal cancers [1–3] - is common, known risk factors such as alcohol use and smoking explain just a fraction of disease causation [4,5] compared to high income settings [6,7]. The fact that ESCC in these regions presents at younger ages [4,8,9], points to multifactorial etiologies with an early age of exposure or the exposure causes more virulent disease. Ingestion of mutagens in diet is a plausible explanation for an exposure with an early age of onset.

Though the International Agency for Research on Cancer (IARC, Lyon, France) classifies red meat as “probably carcinogenic to humans” (group 2A) and processed meat as “carcinogenic to humans” (group 1), [10] to date there is no evidence of red meat as causative of esophageal cancer. For other cancers of the breast, colorectum, and prostate, there is conflicting epidemiological evidence with some studies reporting an increased risk with consumption of well-done cooked meat [11,12], and others have shown no associations [10,13,14]. This uncertainty is partly due to the reliance on self-reports of diet which are prone to measurement error in addition to the recall bias and an inability to disentangle effects from other dietary and lifestyle factors.

Cumulative evidence implicates Heterocyclic Aromatic Amines (HAAs) particularly the 2-amino-1-methyl-6 phenylimidazo [4,5-*b*] pyridine (PhIP) in the pathogenesis of human cancer [15,16]. PhIP and other HAAs are formed during high-temperature cooking (above 150 degrees Celsius) of meat and fish [17]. The formation of PhIP generally increases in meats cooked at higher temperatures or longer duration and dependent on the method of cooking i.e., pan-frying, grilling, or barbecuing produce the highest amounts of PhIP [16,18]. In humans, PhIP undergoes metabolic activation, by cytochrome P450 to form 2-hydroxyamino-1-methyl-6-phenylimidazo [4,5-*b*] pyridine (HONH-PhIP) [19], a genotoxic metabolite that reacts with DNA to form mutation-prone DNA adducts [20].

Scalp hair accrues a portion of the dietary PhIP intake with a linear dose-dependent relationship with PhIP consumed in diet, and it reflects exposure up to several months [20]. In addition, the storage of hair is economical and can be done at room temperature. Thus, hair is a facile and stable specimen to assay dietary PhlP exposure. In order to reduce the burden of esophageal squamous cell carcinoma, identification of risk factors is the first step to the development of targeted interventions. In this study, we aimed to evaluate the association of dietary HAA intake with the PhIP hair biomarker and risk of ESCC in rural southwestern Uganda.

## Methods

### Study design

The polycyclic aromatic hydrocarbon exposures and dietary risk of Esophageal squamous cell carcinoma in southwestern Uganda (PADRE) study is a case-control study that enrolled participants between January 2018 and March 2020 in the endoscopy unit of Mbarara Regional Referral Hospital (MRRH), southwestern Uganda. In this setting, patients with dysphagia present at very late stages (stage III or stage IV) of ESCC disease for upper gastrointestinal endoscopy. To be eligible for the study, ESCC cases and healthy controls had to be 18 years or greater, never have been diagnosed or treated for ESCC. For this study, cases were patients who were diagnosed with ESCC at esophagogastroscopy and esophageal tissue histology.

Controls were healthy community individuals without any gastrointestinal symptoms assessed by the Leeds Dyspepsia Questionnaire and/or normal esophagogastroscopy (EGD) findings. Controls were sampled from the general population at the time of accrual of each ESCC case. Controls were enrolled after interviewing the ESCC patients at their residence. Healthy controls were frequency-matched to ESCC cases by age (≤ 5 years), gender, and area of residence. If there were more than one potential matches, we used the Kish method to select one of them. In the event that a selected matched individual was not home at the time of the visit, we approached the next available individual.

### Data collection procedures

The interviews were conducted in two phases. The first interview was at the endoscopy unit of Mbarara Regional Referral Hospital (MRRH) for ESCC cases. After consenting, esophagogastroscopy was performed to characterize abnormalities in the esophagus and collect esophageal tissue for histology. In addition, a handful (about 25 mg) of participants’ occipital scalp hair was collected from participants with hair. Those with bold heads were instructed not to shave until the second interview where hair was collected. The hair was stored in ziplock bags at room temperature before shipment to the Masonic Cancer Center, University of Minnesota, USA for assay of PhIP.

The second interview was conducted at the participants’ home after histology results were available. Notably, the turn-around time for histology results was 7 days. During this interview, we administered the same questionnaire to ESCC cases and healthy controls to obtain sociodemographic information including age, gender, alcohol drinking, smoking history, source of fuel for cooking, and socioeconomic status based on ownership of household items.

### Dietary assessments

A trained interviewer administered the Diet History Questionnaire II (DHQ II) at the participants’ home. Participants were shown plastic sample dishes as well as drawn images of portion sizes to standardize participants’ understanding of serving size for a period of 12 months. In addition, information on the typical level of doneness and cooking method of red meat commonly consumed in the region i.e., beef, pork, goat, and mutton was obtained. The doneness was qualitatively defined as just until done, well done/crisp, and very well done/charred.

### Quantification of PhIP in hair using ultra performance liquid chromatography electrospray ionization triple quadrupole/tandem mass spectrometry (UPLC-ESI-triple quadrupole (TQ)-MS/MS)

**Figure 1:**
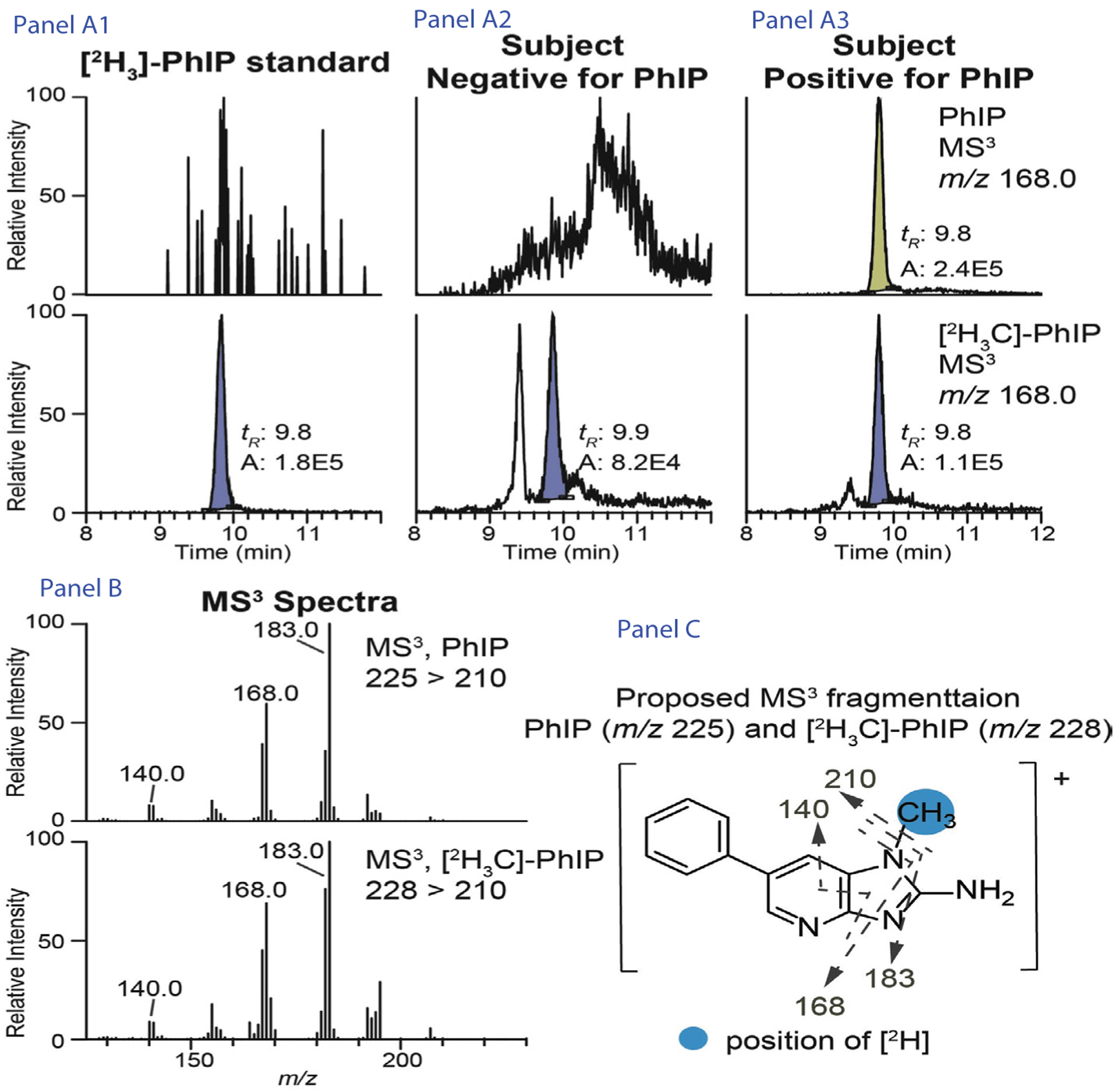
Quantification of PhIP in hair using ultraperformance liquid chromatography electrospray ionization triple quadrupole/tandem mass spectrometry (UPLC-ESI-triple quadrupole (TQ)-MS/MS). Extracted ion current chromatograms shown are from the internal standard [2H3]-PhIP alone (Panel A1), subjects that were negative (Panel A2) and positive (Panel A3) for PhIP. The MS^3^ spectra (Panel B) and the proposed MS^3^ fragmentation of PhIP and [2H3]-PhIP (Panel C) are shown to corroborate the identity of the detected PhIP.

Of note, we did not collect hair from participants who reported dying their hair though there were still dyed hair discovered during assay. The extraction and analysis methods of PhIP from hair have previousely been published [21–23]. (See Appendix text for procedures and Figure 1).

### Data analysis

We reported PhIP levels normalized to the melanin content (ng per g of melanin) instead of crude PhIP levels (pg per g of hair) because the binding affinity of PhIP for eumelanin affects the sequestration of PhIP in hair [24]. Therefore, normalized PhIP is a more accurate measure of PhIP. The limit of quantification (LOQ) value was 26 pgPhIP per g hair. There being no standard cutoff for normal or abnormal PhIP levels, we evaluated the distribution of normalized PhIP by quartitles stratified by ESCC status to determine the quartile where the PhIP levels of ESCC cases differs from that of the controls. Based on this, the normalized PhIP levels were categorized as below 75^th^ percentile and above 75^th^ percentiles.

To estimate the effect of PhIP as potential risk factors for ESCC, we computed adjusted Odds Ratios (aOR) and 95% confidence intervals in multivariable logistic regression models. Covariates of interest were selected based on existing literature including age (each 10 years), gender, smoking (pack years), socioeconomic status, alcohol use (categorized as nondrinker, 1 to 2 drinks per day, 3 to 4 drinks per day, 5 or more drinks per day), and other environmental exposures such as cooking fuel type, location of cooking area. All models were adjusted for age, gender, socioeconomic status, smoking and alcohol use, additional adjusted for cooking fuel and cooking place and lastly adjusted for meat consumption and PhlP. All analyses were performed using Stata version 15.1 (Stata Corp., TX, USA). We determined statistical significance by a 2-sided p-value of less than 0.05.

## Results

Of the 137 hair specimen that were collected from consecutive participants, 20 were not assayed due to the insufficient amounts of hair, and 117 were worked up i.e., cleaned, digested, and PhIP extraction. However, 25 hair samples worked up were found to be brown and thus not assayed by Liquid Chromatography-Mass Spectrometry (LC-MS). Among the 92 samples that were assayed by LC-MS, 3 did not have the melanin content measured though crude PhIP levels were assayed. An additional 7 specimen had interfering ions in Triple quadrupole mass spectrometry (TQ-MS) analysis. Thus, the true and ion interference free sample were 83 for crude PhIP and 85 for normalized PhIP. Table 1 shows baseline characteristics.

**Figure 2:**
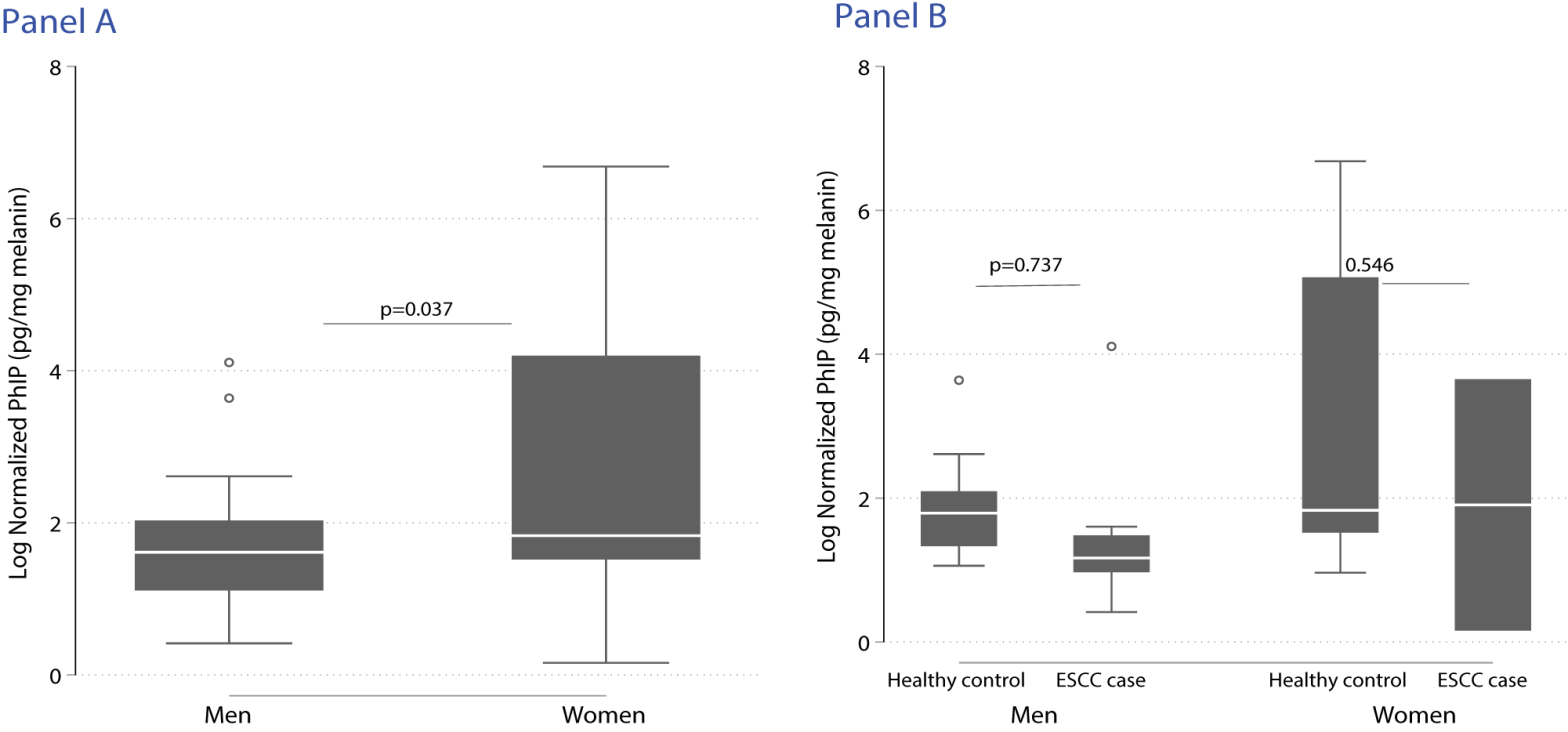
Distribution of log transformed normalized PhIP stratified by gender (Panel A: men vs. women) and ESCC status (Panel B: healthy controls vs. ESCC cases among men and women respectively), PADRE study 2020.

Overall, the mean normalized PhIP (ng/g melanin) was 44.79 (SD 148.08) and higher among healthy controls compared to ESCC cases (58.34 vs. 12.26, p = 0.4). The Figure 2 shows the distributions of log transformed normalized PhIP levels with women having higher normalized PhIP levels compared to men (130.68 vs. 9.00, p = 0.03) The normalized PhIP levels generally were lowest among healthy men (8.31 (SD 8.52) ng/g melanin) and highest among healthy women 158.39 (SD 288.75) ng/g melanin (Table 2).

In the multivariable logistic regression model adjusted for known ESCC risk factors as covariates, the covariates statistically associated with increased odds of ESCC were smoking cigarettes for 2 to 3 pack years (adjusted odds ratios (aOR 4.93, 95%CI 1.48, 16.42), 4 or more pack years (aOR 5.42, 95%CI 1.41, 20.89), drinking 3 to 4 drinks of alcohol per day (aOR 5.92, 95%CI 2.02, 17.39) and 5 or more drinks of alcohol (aOR 5.35, 95%CI 1.03, 27.75) (Table 3 model 1). The estimates were similar when cooking fuel and place were added to the model.

In the fully adjusted multivariable logistic regression model with PhIP measurements, factors associated with increased odds of ESCC included normalized PhIP above 75^th^ percentile (8.65 ng/g of melanin) (aOR4.27, 95%CI 1.12, 16.24) greater odds of ESCC than those with normalized PhIP level lower than the 75^th^ percentile. In addition, ever smoking 2 to 3 pack years of cigarettes resulted in adjusted odds ratio of 7.75 (95% CI 1.90, 31.50) and those 3 or more pack years (aOR5.82, 95%CI 1.25, 27.11). Also, there was an increase in the odds of ESCC with 3 to 4 drinks of alcohol daily (aOR 8.00, 95%CI 2.31, 27.74) (Table 3 model 3).

## Discussion

To our knowledge, this is the first human study reporting an increased risk of ESCC associated with higher scalp hair 2-amino-1-methyl- 6-phenylimidazo [4,5-*b*] pyridine (PhIP) levels. Our finding is important in further elucidating the multifactorial risks of ESCC beyond the known risk factors (alcohol and smoking) that partially explain the disease causation [4,5]. Particularly, dietary risk via ingestion of PhIP that starts early in life is a plausible explanation for the younger age of ESCC presentation in East and Southern Africa [4,8,9]. It is likely that PhIP in synergy with smoking, alcohol, polycyclic aromatic hydrocarbons (PAH), gene-environmental interactions, and other factors are responsible for the increased burden of ESCC in the region. The source of PhIP in our cohort is well-done meat, pork, and poultry, foods widely consumed in sub-Saharan Africa though ESCC burden is highest in East & Southern Africa. However, there exist no other human studies to appraise our findings with.

Of note, the levels of normalized PhIP measured in hair of 44.8 ng/g of melanin in the current study are higher than the levels of PhIP previously detected in hair of participants in other countries e.g., a study of 20 Japanese on their regular diets reported a mean of 16.6 ng/g of melanin [25] and another in 6 non-vegetarians in USA had mean normalized PhIP of 39.42 ng/g of melanin [26]. The differences may be due to multifaceted effects including differences in frequency of exposure, cooking methods or differences in the hepatic cytochrome P450 1A2 (CYP1A2) enzyme which is responsible for PhIP metabolism [27]. The African populations have been described to have lower CYP 1A2 protein content [28,29] and thus higher unmetabolized PhIP in the blood stream which accumulates in the hair follicle. Also, our population was of black race with black pigmentation of hair which confers higher binding affinity for PhIP than in lighter-colored hair [30,31]. In addition, the CYP1A2 enzymatic activity can be influenced by environmental factors such as smoking and diet [32] as well as gene–gene interactions [33]. Together, these differences in PhIP metabolic capacity maybe responsible for the differences in ESCC susceptibility between individuals and geographical regions.

If Phlp is proven as causative, implementable ESCC preventive measures that might have effects include adaption of cooking methods that limit overheating meat to high temperatures will substantially reduce HAA formation and inadvertently PAHs that are also carcinogenic depending on how the meat was cooked and if there are PAHs - if not cooked over a flame or charcoal, the levels of PAHs are lower. There are at least 20 HAAs formed in cooked meats and some of them may contribute to ESCC [16].

Our study has several strengths. The main strength is the use of a well-characterized, population-based case-control study in rural Uganda with high burden of ESCC. Our use of hair PhIP measurement which accurately reflects exposure for at least 6 months [24] exposure misclassification bias compared to self-reported dietary questionnaires.

However, our results should be interpreted with some limitations in mind. First, the observations from this study are limited by the relatively small size of 85 hair specimen assayed for PhIP a single region. Second, all participants were of African ancestry. Therefore, our findings should not be extrapolated to other ethnicities. Lastly, while younger-age individuals living in rural Uganda, future studies are needed to evaluate the generalizability of these findings to other populations in sub-Saharan Africa.

In conclusion, our data offer proof-of-concept that PhIP measurement in hair is a non-invasive and feasible means of assessing PhIP exposure and show an association between PhIP and ESCC in a rural, high ESCC incidence setting. Further study with larger sample with a wider geographical representation is needed to validate hair PhIP for assessment of ESCC risk.

## Figures and Tables

**Table 1:** Baseline characteristics of Esophageal squamous cell carcinoma cases and healthy controls, PADRE study 2020.

Characteristic	Controls n = 54	Cases n = 31
**Demographics**		
Overall age, median (min, max)	59.5 (40, 86)	62 (45, 85)
Age for Men, median (min, max)	59 (45, 85)	60 (45, 84)
Age for Women, median (min, max)	62 (40, 86)	69 (48, 85)
Men, n (%)	33 (61.1)	26 (83.9)
**Asset index** ^§^ **, n (%)**		
Poor	18 (33.3)	10 (32.3)
Fair	20 (37.0)	10 (32.3)
Rich	16 (29.6)	11 (35.5)
**Self-reported family history of cancer**		
Esophageal and stomach cancers	21 (38.9)	14 (45.2)
**Smoking cigarettes, n (%)**		
smoked > 100 cigarettes in lifetime	32 (59.3)	30 (96.8)
Pack years (median, IQR^#^)		
**Alcohol use (times per day), n (%)**		
None	28 (51.9)	8 (25.8)
1 to 2	15 (27.8)	9 (29.0)
3 to 4	9 (16.7)	11 (35.5)
5 or more	2 (3.7)	3 (9.7)
**Primary cooking fuel, n (%)**		
Firewood	50 (92.6)	30 (96.8)
Mean years of primary fuel use	33.8 (11.9)	37.6 (11.3)
**Secondary cooking fuel, n (%)**		
Charcoal	10 (18.5)	7 (22.6)
Years of secondary fuel	15.2 (11.2)	16.8 (13.2)

# IQR: Interquartile range

§ Asset index: calculated from principal components of assets owned

**Table 2:** Distribution of crude and normalized PhIP levels stratified by ESCC status and gender, PADRE study 2020.

Characteristic		All		ESCC Cases		Healthy Controls	
		Men	Women	Men	Women	Men	Women
Crude PhIP level (pg/g hair)	Mean (SD)	199.53 (264.30)	332.38 (555.48)	178.06 (233.39)	807.72 (1100.10)	212.95 (288.52)	142.25 (46.10)
	Median (IQR)	121.90 (82.34, 168.41)	149.92 (65.11, 177.62)	106.19 (71.36, 148.95)	807.72 (29.83, 1585.60)	133.31 (87.72, 189.25)	149.92 (141.15, 177.44)
Normalized PhlP(ng/g melanin)	Mean (SD)	9.00 (13.22)	130.68 (261.42)	10.36 (20.40)	19.83 (26.39)	8.31 (8.52)	158.39 (288.75)
	Median (IQR)	5.02 (3.04, 7.61)	6.25 (4.56, 66.44)	3.22 (2.66, 4.43)	19.83 (1.17, 38.49)	5.99 (3.81, 8.13)	6.25 (4.58, 222.57)

SD: Standard Deviation; IQR: Interquartile range

**Table 3:** Association of PhIP with Esophageal squamous cell carcinoma, PADRE study 2020.

Characteristic	Model 1 Adjusted Odds Ratio (95%CI)	Model 2 Adjusted Odds Ratio (95%CI)	Model 3 Adjusted Odds Ratio (95%CI)
Akaike’s information criterion	123	121	86
Age (each decade in years)	0.79 (0.47, 1.31)	0.79 (0.45, 1.41)	0.82 (0.45, 1.50)
Gender			
Women, n (%)	Ref	Ref	Ref
Men, n (%)	0.33 (0.05, 2.09)	0.29 (0.04, 1.91)	0.46 (0.06, 3.47)
Asset index			
Low	0.64 (0.19, 2.14)	0.51 (0.14, 1.89)	0.43 (0.10, 1.74)
Fair	Ref	Ref	Ref
High	0.73 (0.21, 2.55)	0.57 (0.15, 2.15)	0.68 (0.16, 2.88)
Cigarette smoking (pack years)			
1	Ref	Ref	Ref
2 to 3	4.93 (1.48, 16.42)	6.97 (1.87, 26.01)	7.75 (1.90, 31.50)
3 or more	5.42 (1.41, 20.89)	5.55 (1.30, 23.72)	5.82 (1.25, 27.11)
Alcohol use (number of drinks per day)			
1 to 2 per day	Ref	Ref	Ref
3 to 4 per day	5.92 (2.02, 17.39)	6.55 (2.06, 20.85)	8.00 (2.31, 27.74)
5 or more per day	5.35 (1.03, 27.75)	6.55 (1.07, 39.93)	5.71 (0.89, 36.49)
Primary cooking fuel			
Charcoal		Ref	Ref
Firewood		1.87 (0.03, 25.04)	0.63 (0.01, 20.99)
Cooking place			
Outside in compound		Ref	Ref
Inhouse (separate room)		2.13 (0.13, 35.51)	0.33 (0.01, 11.37)
Inhouse (bedroom)		0.28 (0.01, 9.34)	2.78 (0.15, 49.09)
Meat consumption weekly			
Lower			1.70 (0.43, 6.62)
Middle			Ref
Higher			1.36 (0.31, 5.96)
PhIP normalized (ng/g melanin)			
< 8.65 (75% percentile)			Ref
≥ 8.65 (75% percentile)			4.27 (1.12, 16.24)

CI: Confidence Interval:

## Data Availability

The datasets used and/or analyzed during the current study are available from the corresponding author on reasonable request.

## Appendix Text: Measurement of PhIP

Twenty milligrams of finely minced hair samples were washed three times with 0.1 N HCl followed by CH_3_OH to remove the externally deposited chemicals to avoid false positivity. The hair samples were dried in a ventilated hood for 30 min and digested with 1 N NaOH at 80 °C for 1 hour. The internal standard [^2^H_3_C]-PhIP of 25 pg was added prior to the digestion. The extraction of PhIP was achieved by sequential liquid-liquid extraction with ethyl acetate and solid-phase extraction with SOLA SCX cartridge (Thermo Fisher Scientific, San Jose, CA) [29]. The elutes were dried in a SpeedVac, reconstituted in 30 μL of 5 mM NH_4_HCO_3_ (pH 9.0), and transferred into capLC vials (9 mm, clear vials with fixed inserts, Thermo Fisher Scientific, San Jose, CA). The quantification of PhIP was performed on an Ultimate 3000 RSLC system equipped with a BEH 130 RP18 column (0.3 × 100 mm, 1.7 μm, Waters Corporation, Milford, MS), and a HESI II source interfaced with a TSA Quantiva TQ mass spectrometer (Thermo Fisher Scientific, San Jose, CA). Solvent A was 5 mM NH_4_HCO_3_ (pH 9.0) and solvent B was CH_3_CN. The injection volume was 5 μL. The chromatographic separation was achieved on a linear gradient of 10 to 99 % B in 6 min at 5 μL/min flowrate. MS parameters were: Positive spray voltage 3.3 kV; ion transfer tube temperature 400 °C; vaporizer temperature 70 °C; sheath gas 8 arbitrary unit; auxiliary gas 1 arbitrary unit; dwell time 100 ms; Q1 and Q3 resolution (fwhm) 0.7; CID gas 1.5 mTorr (Figure 1). The selected reaction monitoring (SRM) scan transitions were:PhIP, *m/z* 225.1 > 210.1, 140.1; [^2^H_3_C]-PhIP, *m/z* 228.1 > 210.1, 140.1. The identification of PhIP was confirmed on a UPLC-ESI-linear ion trap (LIT)-MS at the MS^3^ scan stage [44].

Five milligrams of hair samples were digested in Soluene 350:H_2_O (9:1 v/v, 1 mL) (Soluene 350 was obtained from PerkinElmer, Waltham, MA) by heating at 95 °C for 1 hour. Spectra were acquired with a UV/VIS spectrophotometer (Agilent, Santa Clara, CA). The absorbance at 500 nm was used to estimate the total amount of melanin [23].
